# Eyes Offshore: Reporting the Eye Health Needs of Refugees and Asylum Seekers in Australia's Offshore Detention Centres

**DOI:** 10.1111/ceo.14508

**Published:** 2025-04-09

**Authors:** Marcel M. Nejatian, Rosie Dawkins, Ebrar Al‐Yasery, Hessom Razavi

**Affiliations:** ^1^ Lions Eye Institute Nedlands Australia; ^2^ Centre for Ophthalmology and Visual Science The University of Western Australia Crawley Australia; ^3^ Royal Victorian Eye and Ear Hospital East Melbourne Australia; ^4^ Centre for Eye Research Australia Ltd East Melbourne Australia; ^5^ Department of Ophthalmology University of Melbourne Parkville Australia

**Keywords:** asylum seekers, health services accessibility, ophthalmology, refugees

## Abstract

**Background:**

In the last two decades, over 250 000 refugees and asylum seekers have arrived in Australia, many of whom experience significant health problems. Information about their eye health is scarce, particularly for those detained offshore. This is the first study to explore the eye health and services available to this population, helping inform future service planning.

**Methods:**

A mixed‐methods design was employed, incorporating a multicentre audit of an ophthalmology service in Nauru and Manus Island detention centres (2015–2016) and a comparative analysis of this service against established standards of public eye care in mainland Australia. Outcomes included prevalence and causes of vision impairment, management required, and service performance using the Australian Health Performance Framework.

**Results:**

Eighty patients from 14 countries were included (3–57 years, 84% male). There were high rates of bilateral and unilateral vision impairment (19% and 25%, respectively) and bilateral and unilateral blindness (1% and 12%). Bilateral vision impairment was mostly avoidable (80%), with the commonest causes being refractive error and cataract. Ocular trauma accounted for 67% of unilateral blindness. Compared to mainland services, offshore eye care was sub‐standard across all performance domains, including governance, information, workforce, safety, and effectiveness.

**Conclusions:**

Refugees and asylum seekers held in Australia's offshore detention centres had a high burden of eye disease and inadequate access to services. Ceasing offshore detention in favour of onshore processing, as recommended by numerous medical colleges, may help ensure people seeking asylum have access to appropriate eye care in Australia.

## 1 Introduction

In 2022, there were an estimated 35.3 million refugees and 5.4 million asylum seekers worldwide, the highest number in recorded history [1]. Refugees and asylum seekers frequently experience poorer health than the general population [2], including higher rates of vision impairment. Bal et al.'s systematic review identified five studies that reported on the prevalence of vision impairment within refugee camps in North Africa, Eastern Sub‐Saharan Africa, and South Asia [3]. Bilateral blindness, defined by the International Classification of Diseases as a presenting visual acuity (VA) < 3/60 [4], ranged from 4.4% to 26.8% in clinic‐based studies (which recruited individuals with eye symptoms) [5, 6, 7], and from 1.3% to 2.8% in studies screening the general camp population (including asymptomatic individuals) [8, 9]. In comparison, blindness as defined by Australia's less stringent legal criteria (VA < 6/60) affects 0.21% of the general Australian population [10, 11], representing an approximate 10‐fold disparity. Data from Canada corroborates these findings, with a study screening newly arrived Syrian refugees in the community reporting rates of vision impairment which were 32.4 and 13.2 times higher than the general Canadian population, among children and adults, respectively [12, 13]. The available evidence suggests that the main causes of vision impairment among refugees are cataract, uncorrected refractive error, amblyopia, and corneal opacities [7, 8, 9]. There appear to be high rates of conditions which are less common in high‐income countries, including xerophthalmia, trachoma, and traumatic eye injuries [3, 12, 13].

Despite this burden of disease, there is a paucity of research on the eye health of refugees in Australia. Australia has granted permanent protection to over 280 000 refugees and other humanitarian entrants since 2000, with many originating from the Middle East, North Africa, Eastern Sub‐Saharan Africa, and Southeast and South Asia [14]. As with refugees overseas, they may reasonably be expected to experience disproportionally high rates of vision impairment. However, only two Australian studies on this topic exist, both clinical audits of optometry services for refugees [15, 16]. These studies reported bilateral vision impairment rates of 27% (presenting VA < 6/12) [15] and 27.2% (presenting VA < 6/7.5) [16], with nearly 80% of cases in both studies due to uncorrected refractive error.

Furthermore, there are no studies on the eye health of people held within Australia's offshore detention centres on Manus Island and Nauru. Since August 2012, Australia has implemented a policy of sending all asylum seekers arriving by boat to these offshore sites [17]. Offshore detainees are generally considered to experience worse physical and mental health outcomes than those in onshore detention [18, 19, 20, 21], and have documented inadequacies in health care service provision [22, 23, 24]. An analysis of government health reports for offshore detention from 2014 to 2017 indicated a high demand for eye care services [19], with visiting ophthalmologists recruited to meet this demand.

This study provides the first report on the eye health of refugees and asylum seekers (herein referred to as patients) held in Australia's offshore detention centres. A qualitative analysis of services available, compared to public services on the Australian mainland, was also conducted to guide future service planning.

## 2 Materials and Methods

### 2.1 Study Design

**FIGURE 1:**
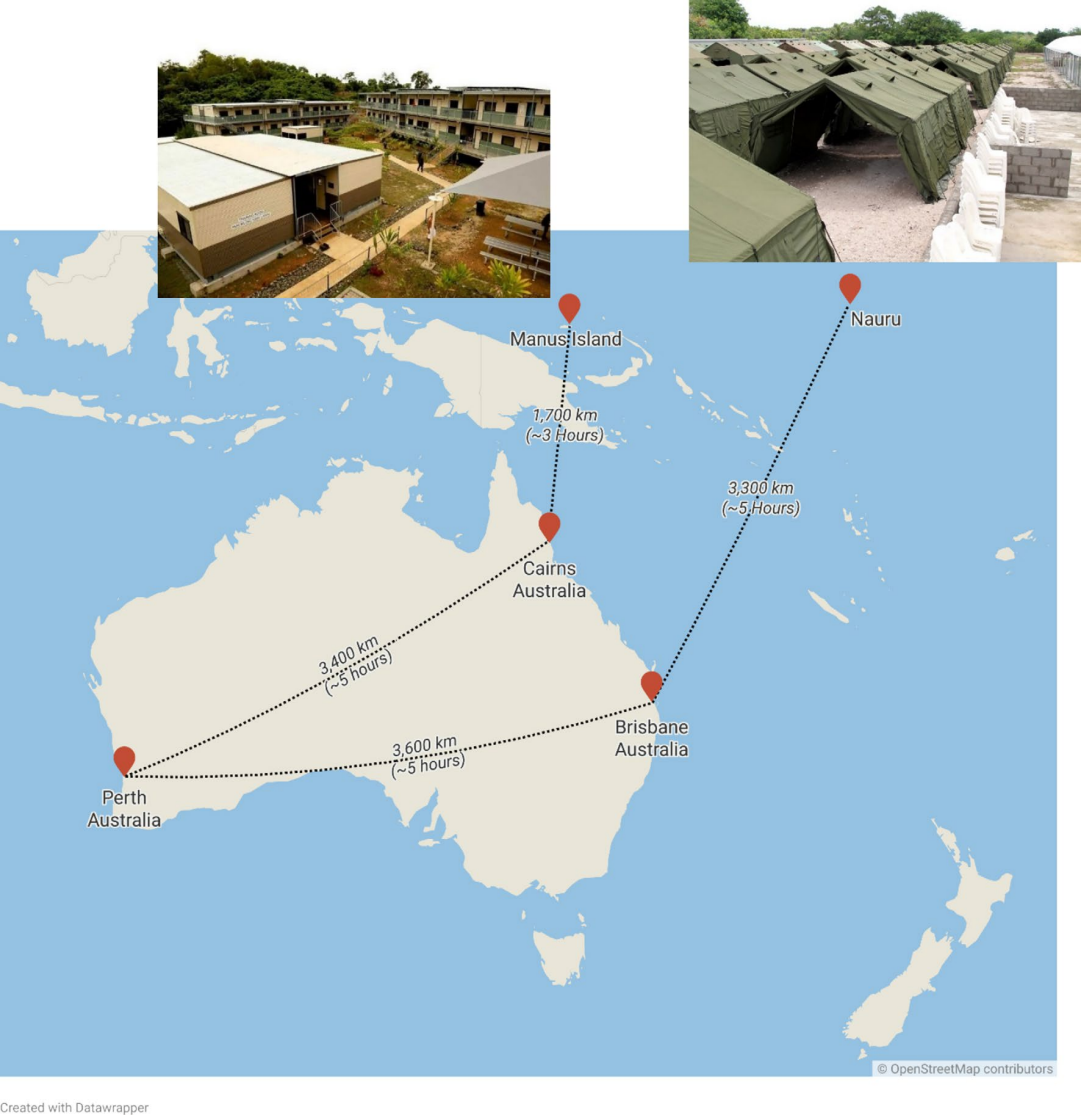
Distances and time travelled by the visiting ophthalmologist to detention centres on Manus Island and Nauru. Source of photos: Australian Government Department of Home Affairs, reproduced under the Creative Commons Attribution 2.0 licence.

A visiting ophthalmologist (H.R.), subcontracted by the International Health and Medical Services Pty Ltd. (IHMS), consulted with patients in offshore detention centres on Nauru (August 2015 and August 2016) and Manus Island (September 2015 and April 2016) (Figure 1). A mixed‐methods study design was employed, comprising a clinical audit of all patient records and a comparative analysis of the service relative to public eye care services in Australia. The study received ethics approval from the Human Research Ethics Committee of The University of Western Australia (2022/ET000334).

### 2.2 Patients and Procedures

Patients were referred to ophthalmology by IHMS medical practitioners or visiting optometrists for symptoms or signs of an ocular condition. The ophthalmologist (H.R.) obtained a history, with interpreters where required, including documented demographics (age, sex, and country of origin) and presenting complaint. Sex was recorded as male or female by the IHMS. Regions of origin were classified using the Global Burden of Disease classification system [25]. Presenting VA with distance spectacles (if worn) was measured at 3 m by H.R. using a Tumbling E‐chart arranged in a conventional Snellen layout, with pinhole testing for VA < 6/12. Intraocular pressures were measured using an iCare tonometer. Subjective refraction for patients previously assessed by an optometrist was recorded. Examination of the anterior and posterior segments was performed by H.R., with pupil dilation (tropicamide 1.0%) where indicated. Diagnoses, management, and follow‐up plans were formulated by H.R.

All demographic and clinical data was prospectively documented in hard and electronic copies by H.R., which were subsequently accessed to obtain study data.

### 2.3 Outcomes

Outcomes of interest were:

1. Prevalence of vision impairment, defined as a presenting VA < 6/12 in one (unilateral) or both (bilateral) eyes, and blindness, defined as a presenting VA < 3/60 [4].
2. The primary cause of vision impairment in each eye as assigned by H.R. When multiple pathologies were present, the most clinically significant was chosen as the primary cause, as per convention [10]. Causes were categorised as avoidable if they could either be treated or prevented by known means.
3. The rates of other ocular conditions diagnosed.
4. The medical, procedural, and/or surgical management and follow‐up for patients.
5. Qualitative comparisons of offshore eye care services to the established standards of public services on the Australian mainland, using the health system input and quality domains of the Australian Health Performance Framework [26].

### 2.4 Statistical Analysis

All analyses were performed using R (V4·1.2; R Core Team, Vienna, Austria). Continuous variables were tested for normality using the Shapiro–Wilk test and presented as mean ± standard deviation if normally distributed or median (quartiles one–three) if non‐normally distributed. Visual acuities were converted from Snellen fractions to LogMAR using the method described by Moussa et al. [27] As visual acuities were not normally distributed, median acuities were reported and expressed in LogMAR (Snellen equivalents rounded to the nearest metre). To explore demographic‐related differences, the prevalence of vision impairment between patients of different sexes, age groups (in 10‐year intervals), regions of origin, and detention centres were compared using the chi‐square test of independence. Missing data was excluded from this analysis. A *p*‐value < 0.05 defined statistical significance.

### 2.5 Comparative Analysis of Eye Care Services

Information on offshore eye care services was obtained through (a) participant (direct, in‐person) observation and documentation performed by the visiting ophthalmologist (H.R.) during visits to Manus Island and Nauru, (b) evaluation of preparatory trip documentation (‘Site Information Guides’) provided to the ophthalmologist (H.R.) by the IHMS prior to visits, and (c) a literature review of documents published to date by the Australian Government and/or IHMS pertaining to offshore health care service provision. The latter documents were identified through searches of the Department of Home Affairs, Parliament of Australia, Federal Register of Legislation, and IHMS websites using the terms ‘Nauru’, ‘Manus Island’, ‘Regional Processing’, ‘Unauthorised Maritime’, ‘Illegal Maritime’, and ‘IHMS’. All information was categorised into the health system input and quality domains of the Australian Health Performance Framework [26], and compared to the standards of mainland service provision set by the Australian Government, Royal Australian and New Zealand College of Ophthalmologists, and the Optometry Board of Australia.

## 3 Results

### 3.1 Patient Demographic and Clinical Characteristics

**FIGURE 2:**
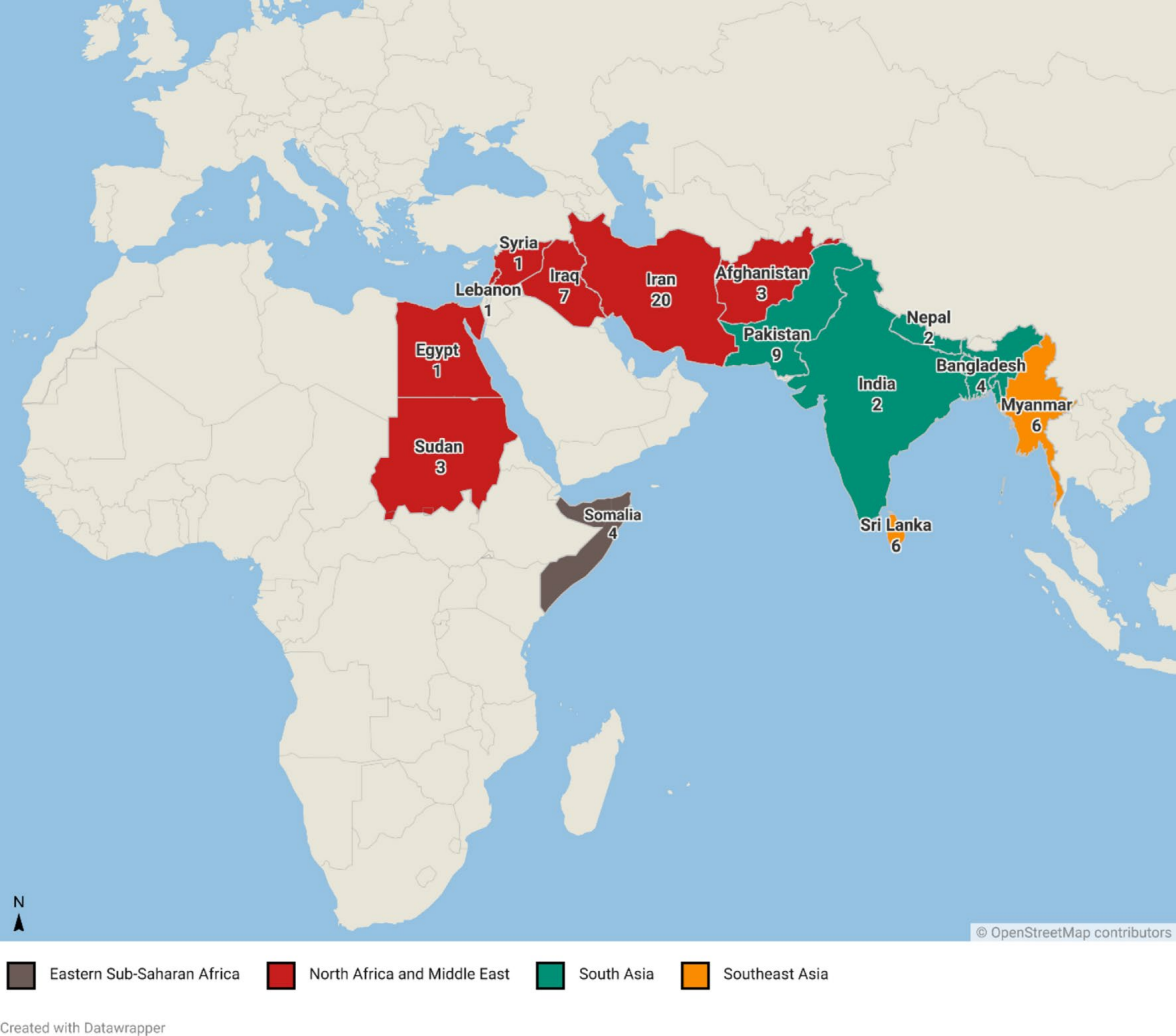
Regions of origin of patients assessed in the Manus Island and Nauru detention centres (*n* = 80).

**FIGURE 3:**
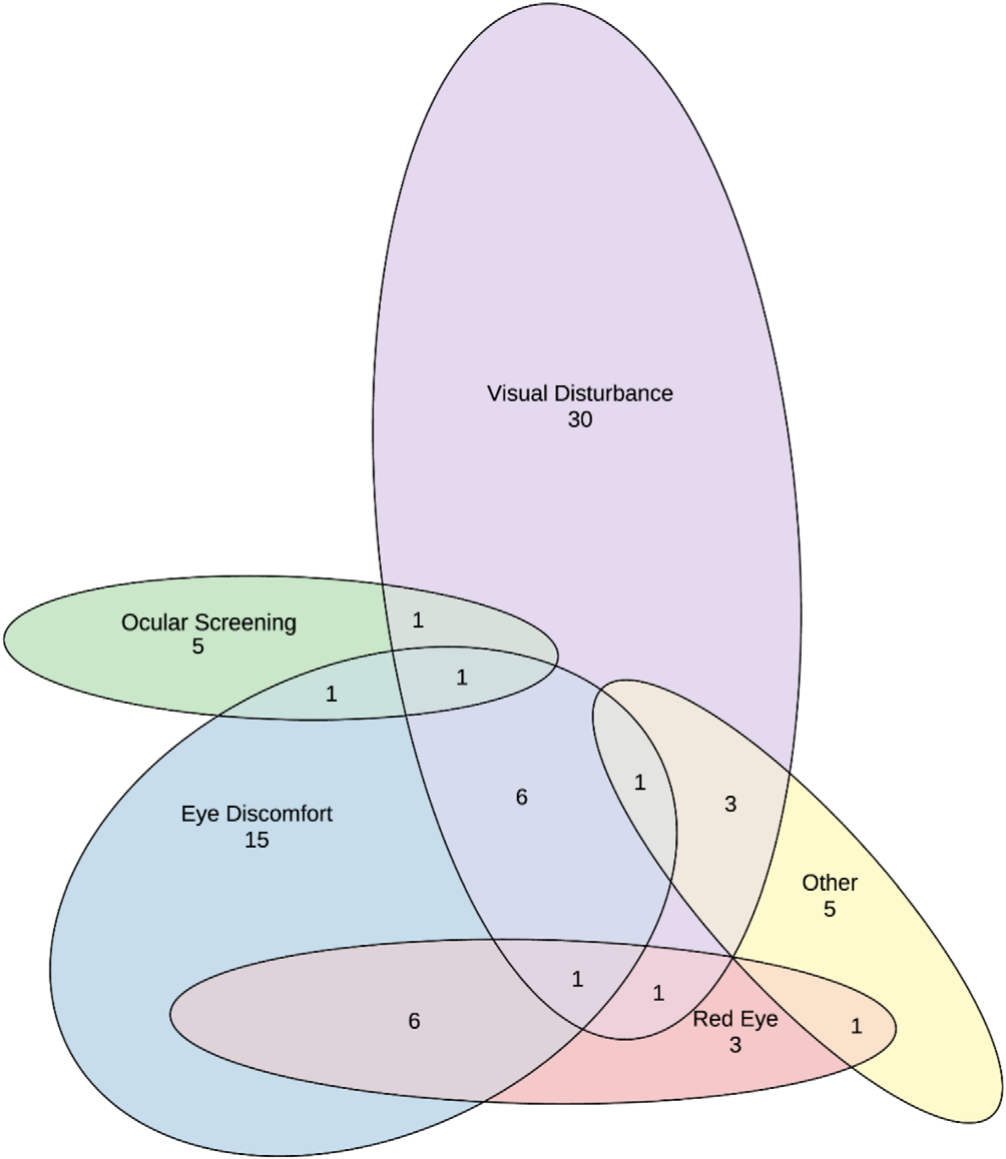
Presenting complaints of patients in Manus Island and Nauru detention centres (*n* = 80). Some patients had more than one presenting complaint.

The sample reported are patients referred with suspected eye disease from a total cohort of 1587 detainees (653 on Nauru and 934 on Manus Island) [28]. A total of 80 patients from 14 countries were assessed following referral to ophthalmology, all of whom were included in the study. Almost half originated from North Africa (*n* = 4) and the Middle East (*n* = 32) (Table 1, Figure 2). The mean age was 34.1 ± 10.7 years. There were two paediatric patients, aged three and seven years. Most patients were male (*n* = 67, 83.8%), and 1 in 8 had diabetes (*n* = 10, 12.5%). The most common presenting complaints were visual disturbance (*n* = 44, 55.0%) and eye discomfort (*n* = 31, 38.8%; Figure 3). Other common reasons for presentation included red eye (*n* = 12, 15.0%) and screening for diabetic retinopathy, glaucoma, or visual field loss from a pituitary tumour (*n* = 8, 10.0%).

**TABLE 1:** Patient demographic and clinical characteristics.

Characteristic	All (*n* = 80)	Manus Island (*n* = 37)	Nauru (*n* = 43)
Age (years)^a^	34.1 ± 10.7	34.4 ± 7.2	33.9 ± 12.7
Female	13 (16.3)	0 (0)	13 (30.2)
Region of origin			
North Africa and Middle East	36 (45.0)	18 (48.6)	18 (41.9)
South Asia	17 (21.3)	10 (27.0)	7 (16.3)
Southeast Asia	12 (15.0)	2 (5.4)	10 (23.3)
Eastern Sub‐Saharan Africa	4 (5.0)	2 (5.4)	2 (4.7)
Undocumented	11 (13.8)	5 (13.5)	6 (14.0)
Diabetes mellitus	10 (12.5)	2 (5.4)	8 (18.6)
Median PVA^b^ (LogMAR, Snellen equivalent)			
Better eye	0.00 (0.00–0.30), 6/6 (6/6–6/12)	0.00 (−0.13–0.35), 6/6 (6/5–6/14)	0.00 (0.00–0.22), 6/6 (6/6–6/10)
Worse eye	0.18 (0.00–0.74), 6/9 (6/6–6/34)	0.10 (0.00–0.65), 6/7.5 (6/6–6/27)	0.19 (0.00–0.78), 6/9 (6/6–6/37)
IOP (mmHg)^c^			
Higher eye	20.2 ± 3.7	19.6 ± 4.1	20.5 ± 3.6
Lower eye	18.3 ± 3.4	17.2 ± 3.8	18.9 ± 3.1

*Note*: Data are presented as mean ± standard deviation, count (percentage), or median (quartiles 1–3).

Abbreviations: IOP = intraocular pressure, PVA = presenting visual acuity.

^a^ Available for 70 patients.

^b^ Available for 77 patients.

^c^ Available for 54 patients.

### 3.2 Vision Impairment

VA was unable to be measured in the two paediatric patients, but they were able to fix and follow targets. One adult had a missing VA in their medical record. VA was documented for the remaining 77 patients. Among these patients, the median VA of the better eye was 0.00 LogMAR (Snellen equivalent 6/6) with an interquartile range of 0.00–0.30 LogMAR (Snellen equivalent 6/6–6/12). The median VA of the worse eye was 0.18 LogMAR (Snellen equivalent 6/9) with an interquartile range of 0.00–0.74 LogMAR (Snellen equivalent 6/6–6/34). The overall rate of bilateral vision impairment was 19.5% (*n* = 15), comprised of 24.3% and 15.0% among patients on Manus Island and Nauru, respectively (*p* = 0.30). A quarter of patients had unilateral vision impairment (*n* = 19). There were no differences in the rate of vision impairment in at least one eye by gender (*p* = 0.83), age group (*p* = 0.12), or region of origin (*p* = 0.60). Many patients had an avoidable cause of bilateral (*n* = 12, 80%) and unilateral (*n* = 9, 47%) vision impairment (Table 2), most commonly from refractive error or cataract. Less common causes of vision impairment included retinitis pigmentosa, adult‐onset vitelliform dystrophy, chorioretinal scarring from Toxoplasmosis, and autoimmune retinopathy (each *n* = 1). Ten patients (13.0%) were blind in at least one eye, seven of whom were under 40 years of age, and seven originated from the neighbouring countries of Iraq, Iran, and Afghanistan. Ocular trauma was the cause of blindness in 7 out of the 11 (64%) affected eyes.

**TABLE 2:** Causes of vision impairment.

Cause	Bilateral	Unilateral
VA < 6/12 to ≥ 3/60	*n* = 14	*n* = 10
Avoidable		
Refractive error	6 (43)	3 (30)
Senile cataract	2 (14)	1 (10)
Amblyopia^a^	2 (14)	1 (10)
Severe allergic conjunctivitis	1 (7)	—
Dry eye	1 (7)	—
Traumatic strabismus	—	1 (10)
Unavoidable		
Retinitis pigmentosa	1 (7)	—
Adult vitelliform dystrophy	—	1 (10)
Chorioretinal scar^b^	—	1 (10)
Not determinable	1 (7)	2 (20)
VA < 3/60 (blind)	*n* = 1	*n* = 9
Avoidable		
Traumatic cataract	—	1 (11)
Traumatic corneal scar	—	1 (11)
Senile cataract	—	1 (11)
Unavoidable		
Other ocular trauma	—	4 (44)
Corneal scar	—	1 (11)
Autoimmune retinopathy	—	1 (11)
Not determinable^c^	1 (100)	—

*Note*: Data are presented as count (percentage).

Abbreviation: VA = visual acuity.

^a^ Two cases of bilateral refractive amblyopia and one case of unilateral amblyopia from a paediatric cataract.

^b^ Prior Toxoplasmosis.

^c^ Right eye blind from traumatic optic neuropathy, undeterminable cause of blindness in left eye.

### 3.3 Other Ocular Conditions

**FIGURE 4:**
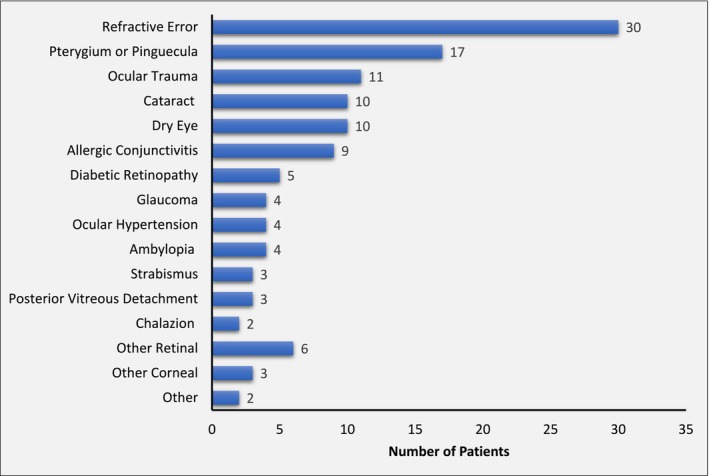
All ocular conditions diagnosed. More than one condition was present in 37 patients. ‘Cataract’ included one case of posterior capsule opacification. ‘Other retinal’ included retinal detachment, epiretinal membrane, retinitis pigmentosa, autoimmune retinopathy, adult‐onset vitelliform maculopathy, and chorioretinal scar. ‘Other corneal’ included keratoconus, corneal scar, and a loose corneal suture. ‘Other’ included episcleritis and punctal occlusion.

Among the 80 patients, the most common ocular conditions diagnosed were refractive error (*n* = 30, 37.5%), pterygium/pinguecula (*n* = 17, 21.3%), and ocular trauma (*n* = 11, 13.8%) (Figure 4). Of the 10 patients with diabetes mellitus, five had diabetic retinopathy, including one with vision‐threatening proliferative disease. Glaucoma was uncommon (*n* = 4, 5.0%) and there were no cases of age‐related macular degeneration.

### 3.4 Management and Follow‐Up Plans

Almost half of all patients were prescribed at least one topical eye drop, including lubricants (*n* = 23), antihistamine and/or decongestants (*n* = 16), steroids (*n* = 7), pressure‐lowering agents (*n* = 3), and antibiotics (*n* = 2) (Table 3). One patient from the Manus Island detention centre was transferred to Port Moresby for pterygium surgery. Thirteen patients (16.3%) who required a procedure or surgery did not receive it, including six with pterygia, five with visually significant cataract (two of whom were unilaterally blind from cataract), one with unilateral blindness from a traumatic corneal scar potentially amendable to a corneal transplant, and one requiring laser treatment for posterior capsule opacification. Over one‐third of patients required review by an optometrist. Forty‐six patients (57.5%) required ophthalmic follow‐up, with 24 requiring lifelong review for chronic conditions such as diabetic retinopathy.

**TABLE 3:** Management and follow‐up plans.

Management and follow‐up	*n* (%)
Topical eye drops or ointment	36 (45.0)
Surgery/procedure performed	
Excision of pterygium and conjunctival autograft	1 (1.3)
I&D of chalazion	1 (1.3)
Removal of corneal suture	1 (1.3)
Awaiting surgery/procedure	
Excision of pterygium and conjunctival autograft	6 (7.5)
Cataract surgery	5 (6.3)
Laser capsulotomy	1 (1·3)
Corneal transplant	1 (1·3)
Other management^a^	3 (3.8)
Referrals	
Optometry	31 (38·8)
Other^b^	6 (7·5)
Ophthalmology follow‐up required	
At least once	46 (57·5)
Regular	24 (30·0)

Abbreviation: I&D = incision and drainage.

^a^ Other management included lid massage and hygiene (*n* = 2) and convergence exercises (*n* = 1).

^b^ Other referrals were to primary care physicians (*n* = 4), endocrinology (*n* = 1), and neurology (*n* = 1).

### 3.5 Comparative Analysis of Eye Care Services

Compared to established standards of public eye care in mainland Australia, offshore services had multiple deficiencies in the health system input domains of the Australian Health Performance Framework (Table 4). While public services on the mainland have transparent, multi‐level governance structures, with published legislation, policies, and clinical guidelines [29, 30, 31, 32, 33, 34, 35], the available online literature revealed no details regarding the governance of service delivery offshore. On the mainland, service audits and published epidemiological research are considered to guide service delivery [32, 35]. In Australia's offshore detention sites, however, direct in‐person observations by the visiting ophthalmologist (H.R.), plus perusal of Site Information Guides and the available online literature indicates that such evidence‐based mechanisms for service planning were, and remain, absent. Mainland public ophthalmology departments have a permanent workforce with expertise in eye care. In contrast, based on direct observation and literature searches, offshore services rely solely on sporadic visits from specialists and lack a resident workforce with eye care expertise [36, 37]. During the study period, offshore sites were directly observed to lack up‐to‐date ophthalmic and imaging equipment found in mainland services, including slit lamps, binocular indirect ophthalmoscopes, tonometers, perimetry, fundus cameras and ocular coherence tomography. They were also observed to lack key ophthalmic pharmaceuticals, such as acetazolamide and topical eye drops for acute angle closure. These deficiencies were confirmed in person by the visiting ophthalmologist with the resident offshore pharmacist during two visits.

**TABLE 4:** Comparative analysis of eye care services provided offshore and on the Australian mainland.

Evaluation component	Mainland eye care	Offshore eye care
Service inputs		
Governance and Structure	Governance structures at federal, state, and local levels including Health Departments, Hospital Executives, and Ophthalmic Heads of Department which collaborate with multiple stakeholders including professional bodies (e.g., The Royal Australian and New Zealand College of Ophthalmologists, Optometry Australia), not‐for‐profit organisations (e.g., Vision 2020, Fred Hollows Foundation), private sectors, universities, and vulnerable groups (e.g., Indigenous communities) [29, 30, 31]. Published standards, policies and guidelines, and institutional arrangements focused on delivery of universally accessible high‐quality services, with monitoring through performance indicators [32, 33, 34, 35]. All public hospitals undergo accreditation to the NSQHS standards at least once every 3 years [32].	Complex arrangement where health care services provided by Nauru or Papua New Guinea, service providers contracted by Australia (e.g., IHMS), or by transfers to Australia and/or third‐party countries. No published details on current governance of such health care provision [36]. Requirement for Independent Health Advice Panel to report on health care standards in place for 9 months before being repealed by the Australian Government in 2019 [37, 38]. No publicly identifiable leadership structure for eye health services. During the study period, the visiting ophthalmologist (H.R.) experienced limited medical autonomy with some clinical decisions subject to non‐transparent review by off‐site administration in Canberra. Similar impediments to medical independence documented by other specialties [39].
Information, Research, and Evidence	All public hospitals must have complete medical records accessible to treating providers and undertake continuous monitoring and improvement in safety and quality of care [32]. Periodic, comprehensive eye health surveys published on national and local levels and used to guide evidence‐based decisions on service provision [35].	During the study period, a ‘health care silo’ model of care was employed, with strict control of patients' clinical information, limited access to past medical history and records from other health care providers. The finding of a non‐transparent offshore environment is corroborated by other disciplines [40, 41]. Quarterly Health Trend Reports with limited information on eye health (only made publicly available after individual requests under the *Freedom of Information Act 1982*) [42]. This includes no explicit information on if and how data is used to guide service provision.
Workforce	Public services with resident medical staff (consultant ophthalmologist/s +/− junior doctors) [43], frequently with other cadres including ophthalmic nurses, opticians, orthoptists, ophthalmic assistants, clerks, and administrative staff. Resident optometry services [43].	During the study period, sporadic visiting eye care specialists with no resident ophthalmologist, optometrist, ophthalmic nurse, optician, orthoptist or ophthalmic assistants. This appears unchanged as per the last Independent Health Advice Panel review in 2019 [36, 37].
Infrastructure	Public services with a suite of basic ophthalmic equipment, theatre, and access to computed tomography or magnetic resonance imaging [44].	During the study period, Lacked all basic ophthalmic equipment including slit lamp, ophthalmoscope, tonometer, pachymeter, retinal camera, ocular coherence tomography, A/B scan biometer, and automated perimetry. No computerised tomography or magnetic resonance imaging. No operating theatre.
Financing	Detailed analysis is beyond the scope of this study	
Service Quality		
Effectiveness	Public services with access to evidence‐based treatments, including medications, ophthalmic lasers, intravitreal treatments, minor procedures, and surgeries [44]. Access to subsidised primary eye care by optometrists and, for certain groups, subsidised spectacles [43]. Access to low vision services for patients who are blind [45].	During the study period, Lacked all basic ophthalmic and imaging equipment, limiting diagnostic and treatment capabilities. No ophthalmic medications for treatment of acute angle closure. Limited capacity for performing minor procedures. No intravitreal treatment, laser, or surgery on‐site. Many referred for spectacles did not receive them. Most (12 out of 13) referred for surgery were not operated on. No low vision services. Documented requests and recommendations regarding clinical effectiveness from the ophthalmologist (H.R.) to the service provider (IHMS) not acknowledged or replied to.
Safety	Public hospitals must undertake organisation‐wide monitoring and reporting of safety outcomes and implement strategies to improve patient safety [32].	No public data on current protocols to monitor and/or improve safety. Documented requests and recommendations regarding clinical safety from the ophthalmologist (H.R.) to the service provider (IHMS) not acknowledged or replied to.
Accessibility	Access to publicly available emergency, clinic, and surgical ophthalmic and optometry services year‐round [43]. Median national waiting time for elective ophthalmic surgery of 105 days [46]. Public access to other medical specialities as required.	During the study period, Emergency services provided by IMHS staff without expertise in eye care or by transfer to local health system. Sporadic visiting ophthalmic and optometry services, with no resident care between visits. Unmet need for surgery (12 out of 13 requiring surgery not being operated on), with no data on wait times. Other medical specialties (e.g., neurology, endocrinology) not accessible or long wait times due to reliance on sporadic visiting services.
Continuity of care	Public hospitals must provide treating providers with access to complete medical records and investigation results and implement structured clinical handovers, when needed, enabling continuity of care [32].	During the study period, Sporadic visits by varying visiting eye specialists with limited access to medical records resulted in fragmented ‘health care silo’ model of care. No provision for visiting ophthalmologist to perform surgery and/or provide post‐operative follow‐up. Little or no information available regarding consults or treatments by off‐site clinicians (located in Papua New Guinea or elsewhere).
Efficiency	The Australian Government and its health jurisdictions are generally not responsible for travel, accommodation, and other incidental costs for doctors working within metropolitan centres. In 2015, total health expenditure per Australian was $7096 [47]. In 2021, the overall cost to the government was $4429 per asylum seeker living in the Australian mainland on a bridging visa [48].	Optometrists and ophthalmologists travelled up to 6900 km for visits during the study period. High travel, accommodation, and incidental costs paid for by the Australian Government, to provide services for a small population. During the study period, there was limited access to medical records leading to duplication of services (e.g., clinic appointments, investigations) and delays in diagnosis and management. In 2015, the Australian Government paid the IHMS $61 510 000 [49] to provide offshore health services for an average number of 1593 detainees [28]—approximately $38 600 per detainee. In 2021, the overall cost to the government was almost $3.4 million per asylum seeker detained offshore [48].
Appropriateness	Public hospitals must have processes in place to seek regular feedback from patients, carers, and families about their experiences and outcomes of care [32].	No readily available information on detainees' satisfaction. Multiple medical specialties have reported offshore care as being inappropriate and unable to meet patients' needs [22, 23, 24].

Abbreviations: IHMS = International Health and Medical Services Pty Ltd, NSQHS = National Safety and Quality Health Service.

Likewise, based on direct in‐person observations and literature searches, the overall quality of offshore eye care was markedly lower than the established standards of mainland services (Table 4). The lack of necessary equipment observed during the study period resulted in patients receiving incomplete assessments relative to those in Australia. The confirmed lack of specific ophthalmic medications (e.g., for angle closure glaucoma or intravitreal treatments) and the lack of a resident workforce limited the ability to treat and monitor both acute and chronic eye conditions. As there was no operating theatre, 12 of 13 (92%) detainees did not receive surgeries recommended by the visiting ophthalmologist, which would be routinely accessible on the mainland [50]. The frequently changing, sporadic workforce, limited availability of medical records, and no clear means of interprofessional communication, as directly observed during the study period, contributed to poorly accessible and fragmented care. For example, while 66 (82.5%) patients required follow‐up with an ophthalmologist and/or optometrist (including one in nine patients with unilateral blindness), there was no indication of whether or when this would occur. Multiple inefficiencies were directly observed and documented by the ophthalmologist (H.R.) during offshore visits, including duplication of services and delays in diagnoses and management. Furthermore, significant staff travel and accommodation requirements (multiple flights and an overnight stay to travel from Australia) amplified costs relative to mainland services. This is supported by the substantially higher government expenditure per patient compared to mainland public health services [28, 47, 49].

## 4 Discussion

This is the first study to document the eye health of refugees and asylum seekers in Australia's offshore detention centres. This group had high rates of vision impairment from diverse ophthalmic pathologies, ranging from simple refractive error to autoimmune retinopathy. Many conditions were unable to be appropriately managed due to the remote location and structural constraints of the offshore health system.

The rate of bilateral vision impairment was 19.5% among the 80 offshore patients referred for ophthalmic evaluation. This is lower than the rates reported in the two available Australian studies of refugees of all ages attending optometry clinics in South Australia (27.0%) and Victoria (27.2%) [15, 16], although the latter study used a more conservative definition of vision impairment (VA < 6/7.5). The rate of bilateral blindness among offshore patients (1.3%) is higher than in the South Australian (0.87%) and Victorian (0.21%) studies, though this should be interpreted with caution given the small number of cases. Similarly, the rate of unilateral blindness among offshore patients (11.7%) is higher than that reported in the Victorian study (1.5%). Direct comparisons of this data with the general Australian population are not possible, as the clinic‐based nature of the available refugee studies would likely inflate the rates of vision impairment compared to a population‐based sample. Future population‐based studies should evaluate whether refugees and the general Australian population experience differences in their eye health needs. Nonetheless, our findings highlight the high burden of vision impairment from complex and varied pathologies among young refugees and asylum seekers previously held in remote detention (mean age 34.1 ± 10.7 years).

Our findings corroborate the existing evidence on the high rates of vision impairment among refugees and asylum seekers internationally. Studies of adult and paediatric refugees presenting to eye clinics in Africa and Asia have consistently reported high rates of bilateral vision impairment (VA < 6/18), ranging from 10.9% to 65.2%, and bilateral blindness (VA < 3/60), ranging from 2.6% to 27.3% [5, 6, 7, 51, 52]. Of these, only one included a comparator group, finding that adult Rohingya refugees attending eye clinics in Bangladesh had significantly higher vision impairment and blindness than non‐refugees [51]. While population‐based studies of refugee populations in Africa and Asia have reported lower rates of bilateral vision impairment (5.4%–12.1%), they still report a high burden of bilateral blindness (1.3%–2.8%) [8, 9, 53]. Contributing factors to the high rates of vision impairment and blindness among these groups may include the harsh conditions and limited access to health services in their home countries, compounded by their hazardous journeys to resettlement [54]. In this study, 7 out of the 11 (64%) cases of blind eyes among patients were caused by trauma, due to exposure to violence, accidents, or other hazards. In contrast, trauma accounts for less than 10% of cases of blindness among the general Australian population [10, 55].

Other diagnoses ranged from common conditions requiring primary eye care (e.g., refractive error and dry eye) to complex pathologies requiring tertiary care (e.g., retinitis pigmentosa, autoimmune retinopathy, and a corneal scar requiring a corneal transplant). Of note, there was a high proportion of avoidable vision impairment, including 80% of bilateral vision impairment that was treatable through spectacles, cataract surgery, or eye drops for ocular surface disease. This finding is consistent with other studies of refugee and asylum‐seeker groups worldwide [7, 8, 9, 15, 16, 51, 53]. Concerningly, several patients for whom spectacles were requested did not receive them during the study period, indicating a lack of basic primary eye care.

The comparative analyses suggest that relative to mainland services in Australia, offshore eye care was sub‐standard across all 11 health system input and quality domains of the Australian Health Performance Framework. Gaps in health service inputs, including inadequate governance, information and research systems, workforce, and infrastructure may be expected to contribute to the deficiencies noted in the quality of eye care relative to mainland services. Observed deficiencies included less effective, safe, accessible, continuous, and appropriate care relative to mainland services. This is despite the available financial data indicating that health care offshore has cost significantly more than health services available in mainland Australia [28, 47, 49].

These deficiencies existed despite the government's commitment to providing offshore detainees with ‘access to health care to a level, standard and timeliness broadly consistent with health care available to the Australian community’ [56, 57]. These findings corroborate numerous reports of inadequate provision of other health services, including mental health [22], primary care [23], obstetrics [23], sexual health [24], paediatrics [22, 24], emergency care [22], and infectious diseases [22, 23]. This report adds to the growing medical evidence that suggests the health care needs of refugees and asylum seekers cannot be adequately met in offshore detention centres. While the Manus Island detention centre was closed in October 2017, the policy of offshore detention on Nauru persists, as evidenced by the transfer of at least 100 asylum seekers from Australia to Nauru in the last year [58].

In the setting of current global conflicts, refugee numbers are set to rise beyond already record‐high levels [1], requiring robust and sustainable solutions for the provision of health care to people seeking asylum. Onshore processing may facilitate their access to more appropriate mainstream primary, secondary, and tertiary‐level eye and other health care services. This approach aligns with recommendations from the Royal Australian and New Zealand College of Ophthalmologists [59] and other medical colleges [60, 61, 62, 63].

### 4.1 Limitations

Our sample of 80 patients was drawn from a total cohort of 1587 detainees (653 on Nauru and 934 on Manus Island), representing approximately 5.0% of the entire detainee population [28]. This appears lower than the proportion of refugees who accessed optometry services after on‐arrival health screens in South Australia (494 out of 1400, or 35.3% in a recent audit) [15], suggesting the potential under‐referral of patients in offshore centres. While the clinic‐based nature of the sample may limit the generalisability of our findings, our study provides valuable insight into the presenting eye conditions among this population. To our knowledge, it is the first ophthalmic study of its kind, despite Australia's longstanding policy of offshore detention spanning over two decades. Furthermore, numerous barriers would likely obstruct a population‐based survey of the offshore cohort, such as the repeated denial of visas for researchers and human rights advocates [64] and, prior to August 2017, the Australian Border Force Act 2015, which criminalised the disclosure of ‘protected’ information by detention centre staff [65]. Importantly, the data in this study will help address the lack of literature on refugee eye health, a gap identified by the Royal Australian and New Zealand College of Ophthalmologists [59].

Despite data being collected in 2015–2016, our findings are especially relevant now, given the recent surge in the number of asylum seekers sent to Nauru by the Australian Government [58]. However, our study may not reflect any potential unannounced changes to offshore services which have occurred since 2016, of which we are unaware. Delays in data analysis were due to limited personnel resources and potential legal constraints under the Australian Border Force Act 2015, which, until its amendment, restricted the disclosure of health information from offshore detention centres [65]. While a comparator group would have allowed statistical analysis of differences between refugee and non‐refugee eye health, no suitable group existed as all offshore detainees were from a refugee background. The comparative health systems analysis was predominately descriptive and would benefit from the inclusion of performance indicators such as waiting times, non‐attendance rates, measures of patient satisfaction, and cost per appointment. These data are not publicly available, but efforts could potentially be made to seek them through Freedom of Information applications for future research. Future studies could incorporate a strengths‐based (rather than deficit‐based) approach to identify resilience factors in how participants manage their eye health [66] and, if possible, co‐produce the research with representative members of the study population [67].

### 4.2 Conclusion

This is the first study to evaluate the eye health of refugees and asylum seekers in Australia's offshore detention centres. This population had a high burden of vision impairment from diverse ophthalmic conditions, with a high proportion of cases being avoidable. There were widespread, systemic concerns with the quality of eye care services available offshore, adding evidence to the call from Australia's medical community to close offshore detention centres in favour of onshore processing on the Australian mainland.

## Conflicts of Interest

The authors declare no conflicts of interest.

## Data Availability

Access to the original, de‐identified study data and a data dictionary can be obtained upon reasonable request to the corresponding author. Data will be shared through providing access to the investigators' Institutional Research Data Store (IRDS) at The University of Western Australia.
